# Romiplostim for refractory chemotherapy-induced thrombocytopenia in a long-term survivor of limited-stage small cell lung cancer: a case report

**DOI:** 10.3389/fonc.2026.1854366

**Published:** 2026-09-17

**Authors:** Mingfang Wang, Ping Tian, Taosheng Huang, Kai Sun, Jie Liu, Peng Wang

**Affiliations:** Yidu Central Hospital of Weifang, Weifang, China

**Keywords:** chemotherapy-induced thrombocytopenia, refractory thrombocytopenia, romiplostim, small cell lung cancer, thrombopoietin receptor agonist

## Abstract

**Background:**

Aggressive chemoradiotherapy for limited-stage small cell lung cancer (SCLC) frequently causes severe hematologic toxicities, particularly chemotherapy-induced thrombocytopenia (CIT), which can compromise treatment efficacy. While thrombopoietin receptor agonists (TPO-RAs) are preferred for CIT management, refractory cases pose significant challenges.

**Case presentation:**

A patient with stage IIIC (cT3N3M0) limited-stage SCLC achieved a durable partial response exceeding five years following etoposide/cisplatin chemotherapy and concurrent thoracic radiotherapy (60 Gy). However, severe, persistent, and refractory CIT developed, unresponsive to multiple interventions including recombinant human thrombopoietin (rhTPO), avatrombopag, eltrombopag, corticosteroids, thalidomide, tretinoin, and intermittent platelet transfusions. Bone marrow aspiration confirmed profound hypocellularity and megakaryocytic hypoplasia.

**Intervention & outcome:**

Initiation of subcutaneous romiplostim successfully elevated and maintained platelet counts, enabling sustained disease control. Platelet counts declined rapidly below 100×10&xxx2079;/L upon extending the dosing interval beyond 3-4 weeks, indicating that platelet maintenance remained dependent on continued romiplostim administration.

**Conclusion:**

This case demonstrates romiplostim’s efficacy in managing severe, refractory CIT following intensive SCLC therapy, facilitating long-term disease control in a sustained responder. It highlights the critical role of TPO-RAs in this setting but underscores challenges including ongoing dependence on continued romiplostim for platelet maintenance and the need for strategies to coordinate romiplostim with future systemic therapies should disease progression occur. The potential for loss of romiplostim response necessitates exploration of alternative approaches.

## 1 Introduction

Small cell lung cancer (SCLC) is an aggressive malignancy characterized by rapid growth and early metastasis, with over 70% of patients presenting with extensive-stage disease at diagnosis (1). For limited-stage SCLC, the cornerstone of first-line treatment is platinum-based chemotherapy (typically etoposide plus cisplatin, EP) combined with thoracic radiotherapy, an approach that has been further enhanced by the integration of immune checkpoint inhibitors like durvalumab (2, 3). However, the intensification of this multimodal therapy inevitably amplifies treatment-related toxicities, particularly hematological adverse events. Severe myelosuppression, including neutropenia and thrombocytopenia, occurs frequently, affecting more than 30% of patients receiving chemoradiotherapy, and represents a major cause of treatment delays or dose reductions that can compromise efficacy (4).

Chemotherapy-induced thrombocytopenia (CIT) poses a significant clinical challenge due to the risk of bleeding and the historical lack of broadly effective pharmacological interventions (5). While recombinant human granulocyte colony-stimulating factor (rhG-CSF) has improved the management of neutropenia, effective strategies for CIT have been more elusive. Interleukin-11 (IL-11) was previously explored but is no longer recommended due to significant drawbacks, including dose-dependent fluid retention, cardiotoxicity, arrhythmias, and critically, well-documented pro-oncogenic properties that may promote tumor growth, metastasis, and chemoresistance (6).

Thrombopoietin receptor agonists (TPO-RAs) have emerged as the preferred therapeutic class for managing CIT. These agents stimulate platelet production by activating the thrombopoietin receptor (c-Mpl) on megakaryocytes (7, 8). Recombinant human thrombopoietin (rhTPO) demonstrated efficacy but faced potential limitations like immunogenicity. Second-generation TPO-RAs, including the peptibody romiplostim and oral agents like avatrombopag (9), eltrombopag (10), and hetrombopag (11), offer distinct pharmacological profiles and administration routes (8). Romiplostim, administered subcutaneously, exhibits potent thrombopoietic activity through molecular targeting of c-Mpl, activating key signaling pathways (JAK2/STAT5, PI3K/Akt, ERK), and uniquely, beneficial immunomodulatory effects via TGF-β enhancement and potential Fc-mediated immune tolerance (12).

This report details the case of a patient with limited-stage SCLC (cT3N3M0, stage IIIC) who achieved a durable partial response (PR) exceeding five years following treatment with concurrent chemoradiotherapy (EP regimen plus 60 Gy radiotherapy). However, the patient developed severe, persistent, and refractory CIT, unresponsive to multiple therapeutic interventions including rhTPO, avatrombopag, eltrombopag, corticosteroids, thalidomide, tretinoin, and intermittent platelet transfusions. Bone marrow aspiration confirmed profound hypocellularity and megakaryocytic hypoplasia. Ultimately, romiplostim therapy successfully elevated and maintained platelet counts, enabling long-term disease control. This case highlights the critical role of TPO-RAs, specifically romiplostim, in managing refractory CIT within the context of aggressive SCLC therapy, while also underscoring the challenge of ongoing dependence on continued romiplostim for platelet maintenance and the need for strategies to coordinate CIT management with potential future systemic anticancer treatments should disease progression occur.

## 2 Case presentation

### 2.1 Chief complaint

A 55-year-old man, previously a farmer, presented with cough and expectoration for over one month. No headache, dizziness, nausea, or vomiting. No hoarseness, dysphagia, shortness of breath, or chest pain. No hemoptysis or limb weakness.

### 2.2 Past medical history

Previously healthy.

### 2.3 Personal history

Smoking history of 60 pack-years (1200 cigarette-years), quit approximately 1 month prior to diagnosis. Alcohol consumption history for over 30 years, equivalent to approximately 212 grams per day. Family history was unremarkable for malignancy. His past medical history is negative for any chronic diseases, previous surgeries, or severe trauma.

### 2.4 Physical examination

Zubrod performance status score of 1. Scattered skin petechiae present over the body, more pronounced on both lower limbs and back. No superficial lymphadenopathy detected. Lungs clear without rales or rhonchi. Heart rhythm regular; no pathological murmurs auscultated. Abdomen soft and flat, no tenderness or rebound tenderness. Liver and spleen not palpable below costal margins. No costovertebral angle tenderness. No abnormal abdominal masses palpated. No mucosal, gingival, nasal, conjunctival, or gastrointestinal bleeding was present.

### 2.5 Imaging studies

Contrast-enhanced Chest CT: A left hilar mass measuring approximately 5 × 7 cm, encasing the left upper lobe bronchus. Multiple enlarged lymph nodes noted in the mediastinum and left hilum; the largest, located anterior and right to the trachea, measured approximately 3.7 × 4.6 cm.

Bone Scan (ECT):No definite evidence of bone metastasis.

Contrast-enhanced Brain MRI: No evidence of intracranial metastasis.

Neck Ultrasound: No significant cervical lymphadenopathy detected.

### 2.6 Laboratory investigations

Final Diagnosis: Small cell lung cancer of the left lung (cT3N3M0 stage IIIC, limited stage). Metastases to bilateral mediastinal and bilateral hilar lymph nodes(**Figure 1**).

**Figure 1:**
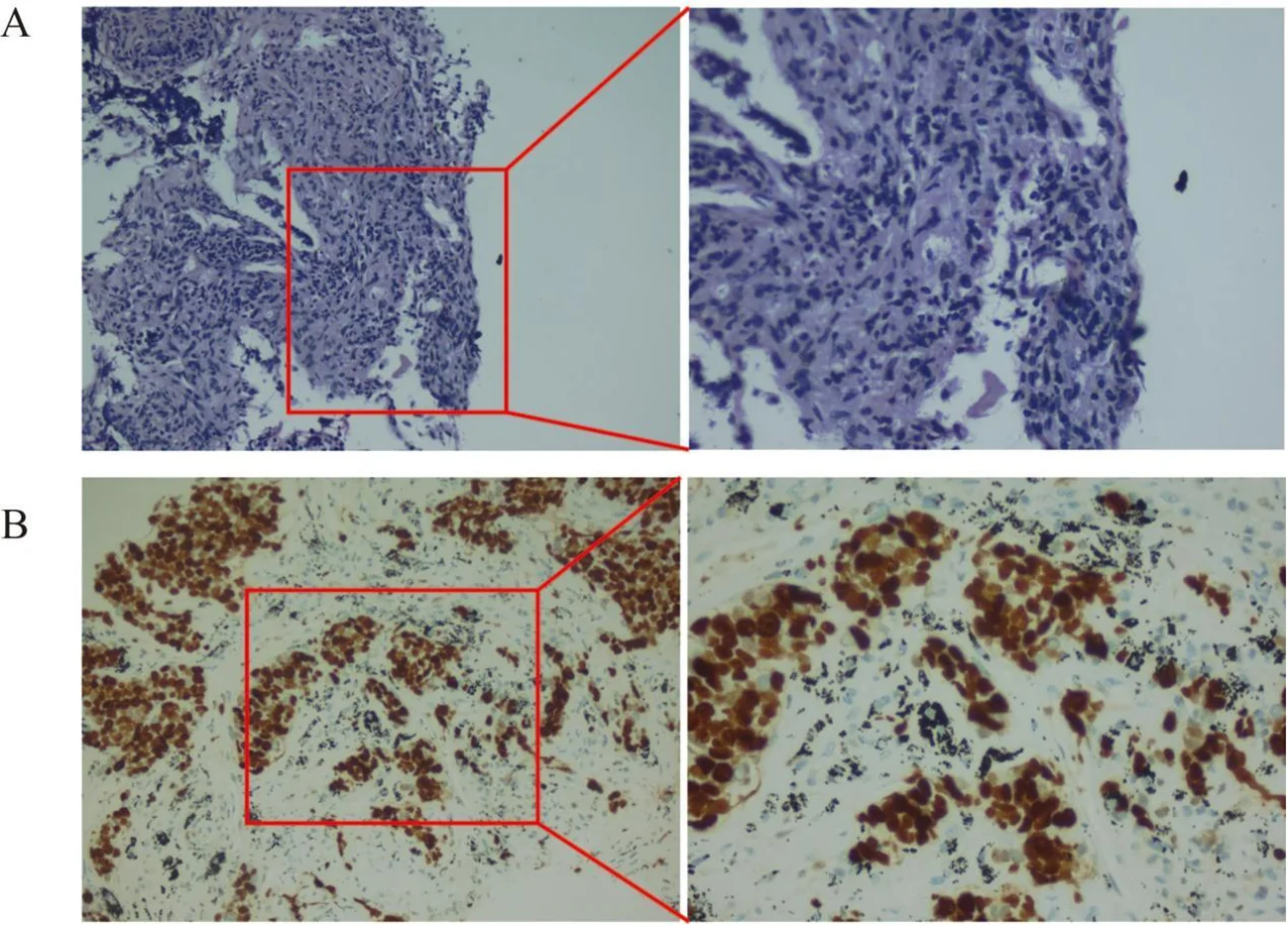
Histopathological confirmation of small-cell lung cancer. **(A)** Haematoxylin–eosin staining shows small tumour cells with scant cytoplasm and finely granular chromatin. **(B)** Immunohistochemistry shows a high Ki-67 proliferation index.

### 2.7 Treatment course

Cycle 1-4: EP regimen (cisplatin 75 mg/m² on day 1 plus etoposide 100 mg/m² on days 1–3, every 21 days, per the CONVERT protocol (18)) for 4 cycles.

Cycle 5: Concurrent chemoradiotherapy with EP regimen.

Radiotherapy regimen: A total dose of 60 Gy in 30 fractions.

Cycle 6: EP regimen (Etoposide + Cisplatin) for 1 cycle.

### 2.8 Treatment response evaluation

Partial Response (PR)

Treatment response evaluations after cycles 2, 4, and 6: PR.

Re-evaluation at 90 days post-treatment completion: PR.(**Figure 2**)

**Figure 2:**
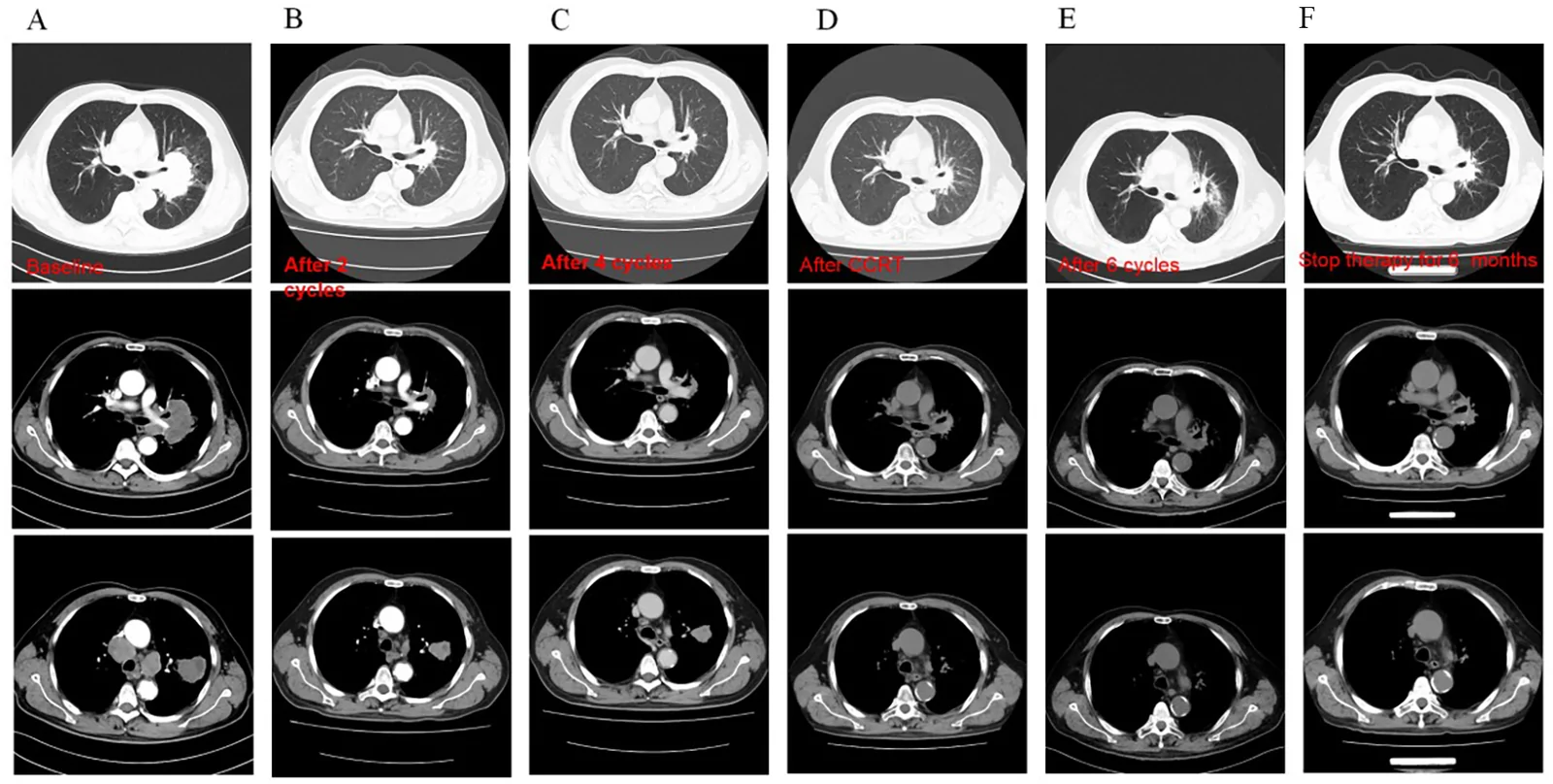
Serial contrast-enhanced chest CT. **(A)** Baseline; **(B)** after 2 cycles of chemotherapy; **(C)** after 4 cycles; **(D)** after concurrent chemoradiotherapy; **(E)** after 6 cycles; **(F)** 6 months after treatment completion. The tumour maintained a partial response throughout.

### 2.9 Treatment adverse effects

#### 2.9.1 Neutropenia

Grade 3 neutropenia occurred during cycle 2 (WBC: 2.61×10^9^/L, ANC: 0.65×10^9^/L, Platelets: 235×10^9^/L). Corrected after administration of G-CSF (6 mg). Subsequent cycles included prophylactic pegylated recombinant human G-CSF (6 mg) administered 24 hours post-chemotherapy, preventing further severe bone marrow suppression.

#### 2.9.2 Severe neutropenia and thrombocytopenia

Post-Treatment: Grade 4 neutropenia developed after chemoradiotherapy completion (WBC: 0.59×10^9^/L, ANC: 0.15×10^9^/L, Hemoglobin: 94 g/L, Platelets: 27×10^9^/L) accompanied by fever (peak temperature >40 °C) and scattered skin petechiae. Managed with G-CSF, antibiotics, IL-11 (interleukin-11), and cafaminol (coagulation stimulant). Neutropenia resolved; thrombocytopenia persisted.

#### 2.9.3 Thrombocytopenia management summary

##### 2.9.3.1 Phase 1

Treatment: Oral avatrombopag (20 mg once daily) or eltrombopag (25 mg once daily) (approximately 4–13 months after diagnosis; throughout, time points are expressed relative to the date of diagnosis).

Outcome: Ineffective; no significant platelet increase.

##### 2.9.3.2 Phase 2

Treatment: Prednisone (1 mg/kg/day) + thalidomide (50 mg nightly) + tretinoin (all-trans retinoic acid, 10 mg twice daily) (approximately 13–15 months after diagnosis).

Outcome: Ineffective; no significant platelet increase.

##### 2.9.3.3 Phase 3

Treatment: Prednisone (1 mg/kg/day) + thalidomide (50 mg nightly) + tretinoin (all-trans retinoic acid, 10 mg twice daily) + recombinant human thrombopoietin (rhTPO) 15000 units/day for 14 days (approximately 15–20 months after diagnosis).

Outcome: Ineffective; no significant platelet increase.

Perform a bone marrow aspiration test: (approximately 20 months after diagnosis)

The bone marrow cellularity is markedly decreased, nucleated cells are sparse, fat cells are significantly increased presenting a “desolate” sign; hematopoietic cells of three lineages are concurrently reduced, the proportion of erythroid precursors at all stages sharply decreases, precursor granulocytic cells and mature neutrophils are significantly reduced and a developmental “gap” can be seen, the number of megakaryocytes is markedly reduced, platelet production is extremely low;the morphology of residual cells is normal, no increase in blast cells, no dyshematopoiesis or malignant cell infiltration;non-hematopoietic components in the interstitium are relatively increased.(**Figure 3**)

**Figure 3:**
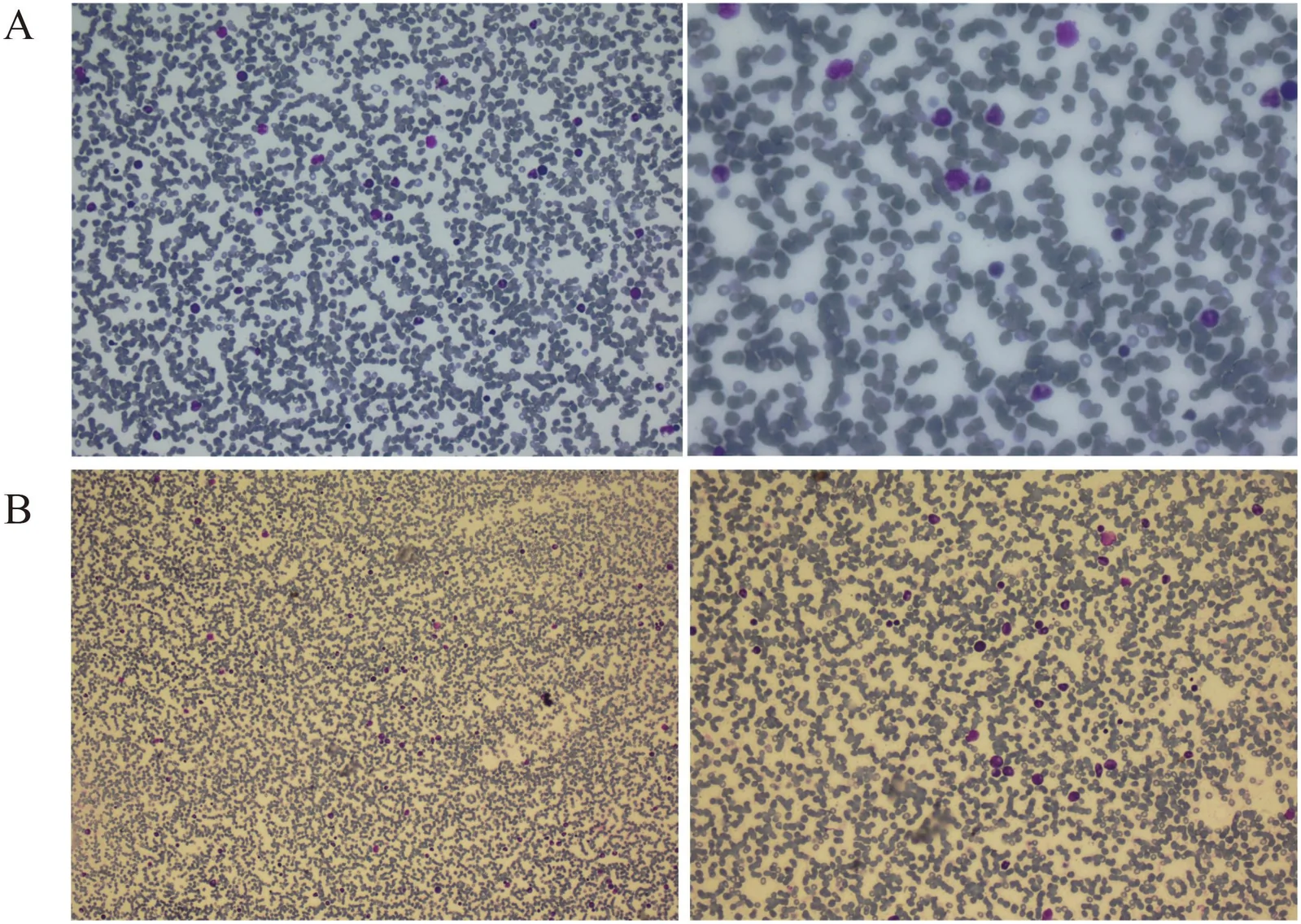
Bone marrow aspiration. **(A)** The aspirate smear shows severe hypocellularity, with markedly reduced nucleated cells and increased adipose tissue. **(B)** All three lineages are suppressed — scant erythroid precursors, granulocytic maturational arrest, and near-absent megakaryocytes; the residual cells are morphologically normal, with no blasts, dysplasia or malignant infiltration.

##### 2.9.3.4 Phase 4

Treatment: Intermittent platelet transfusions (approximately monthly) plus rhTPO (from approximately 20 months onward).

Outcome: Ineffective. Platelet counts transiently increased post-transfusion but declined to <10×10^9^/L within 3–4 weeks.

##### 2.9.3.5 Phase 5

Treatment: Romiplostim 250 µg weekly (qw), later changed to Romiplostim 250 µg every 3 weeks (q3w) approximately 57 months after diagnosis [≈4.8 years] to present.

Outcome: Effective. Platelet counts increased significantly (up to 263×10^9^/L) within 1 week of administration. However, counts decreased below 100×10^9^/L upon extending the dosing interval.

### 2.10 For now

The patient maintains well-controlled cancer and has been in a partial response (PR) state for over 5 years. The patient receives romiplostim via subcutaneous injection every three to four weeks for thrombocytopenia. The all-treatment plan is showed in **Figure 4** and **Table 1**.

**Figure 4:**
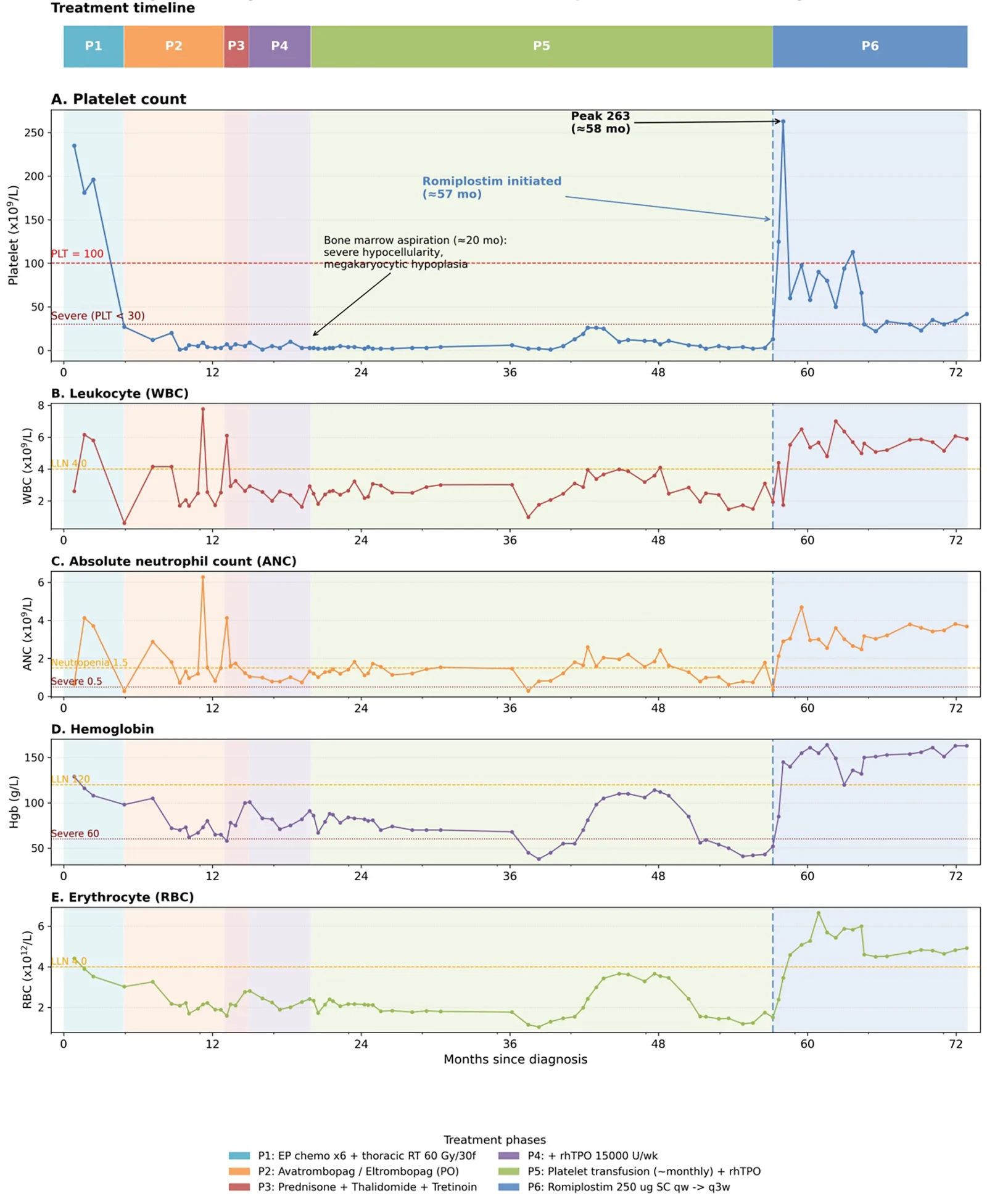
Longitudinal blood counts and treatment phases. Platelet **(A)**, leukocyte **(B)**, neutrophil **(C)**, haemoglobin **(D)** and erythrocyte **(E)** counts across the six treatment phases (P1–P6, top ribbon). Romiplostim (dashed line, ~57 months after diagnosis) raised the platelet count to a peak of 263×10^9^/L, which then declined when the dosing interval was extended.

**Table 1:** Chronological summary of thrombocytopenia management, platelet response, and outcome.

Phase	Period	Intervention (dose / duration)	Platelet (×10^9^/L)	Response / outcome
P1	Diagnosis to ~5 months	EP chemotherapy ×6 cycles (cisplatin 75 mg/m² d1 + etoposide 100 mg/m² d1–3, q21d) + concurrent thoracic radiotherapy 60 Gy/30 fractions; prophylactic PEG-G-CSF 6 mg from cycle 2	Baseline 235 → post-treatment nadir 27	Grade 4 neutropenia (WBC 0.59) and severe thrombocytopenia emerged; neutropenia resolved, thrombocytopenia persisted
P2	~5–13 months	Avatrombopag 20 mg once daily or eltrombopag 25 mg once daily (oral)	1–27 (persistently low)	Ineffective; no significant platelet increase
P3	~13–15 months	Prednisone 1 mg/kg/day + thalidomide 50 mg nightly + tretinoin (ATRA) 10 mg twice daily	3–7	Ineffective
P4	~15–20 months	Prednisone 1 mg/kg/day + thalidomide 50 mg nightly + tretinoin 10 mg twice daily + rhTPO 15000 U/week	1–10	Ineffective
—	~20 months after diagnosis	Bone marrow ASPIRATION (no biopsy / flow / cytogenetics / PNH / molecular performed)	—	Severe hypocellularity, trilineage suppression, markedly reduced megakaryocytes; no dysplasia or malignant infiltration
P5	~20–57 months	Intermittent platelet transfusion (~monthly) + rhTPO	1–26	Ineffective; transient post-transfusion rise, declined to <10 within 3–4 weeks
P6	~57 months to present	Romiplostim 250 µg subcutaneously, weekly (qw) → every 3 weeks (q3w)	13 → peak 263; falls <100 when interval extended	Effective; platelet maintained, but maintenance dependent on continued romiplostim administration

## 3 Discussion

Small cell lung cancer (SCLC) accounts for approximately 15% of lung cancers, and over 70% of patients present with extensive-stage disease (1, 13, 14). For limited-stage disease, platinum–etoposide chemotherapy combined with thoracic radiotherapy is the cornerstone of first-line treatment, and the addition of immune checkpoint inhibitors (e.g., the ADRIATIC trial) further improves survival (10, 15). This multimodal intensification, however, amplifies treatment-related toxicity — grade ≥3 neutropenia affects >30% of patients (16) — underscoring the need for strategies that limit toxicity while preserving efficacy (17).

Concurrent chemoradiotherapy significantly exacerbates myelotoxicity, particularly myelosuppression (15, 18). This manifests primarily as neutropenia, anemia, and thrombocytopenia. Although supportive care measures, including recombinant human granulocyte colony-stimulating factor (rhG-CSF), have substantially ameliorated chemotherapy-induced neutropenia, thrombocytopenia remains a persistent challenge. Thrombocytopenia is particularly problematic as it lacks broadly effective pharmacological interventions and represents a leading cause of chemotherapy dose delays or interruptions. Effective management of chemotherapy-induced thrombocytopenia (CIT) is therefore crucial for maintaining treatment intensity and optimizing outcomes in SCLC (19).

Historically, interleukin-11 (IL-11) was used as a thrombopoietic agent for CIT, promoting megakaryocyte maturation and platelet release through JAK-STAT signalling (20, 21). However, its use is now discouraged: beyond dose-dependent fluid retention and atrial arrhythmias, IL-11 has well-documented pro-oncogenic properties — persistent STAT3 activation promotes tumour proliferation and chemoresistance, and IL-11 derived from cancer-associated fibroblasts drives tumour growth and correlates with poor prognosis (22, 23). Given this toxicity and pro-tumorigenic potential, IL-11 is no longer recommended for CIT management.

Thrombopoietin receptor agonists (TPO-RAs) are now the preferred approach for CIT (24). A 2025 network meta-analysis (16 RCTs, n=1,746) confirmed that they significantly reduce chemotherapy delays or dose reductions (OR = 0.37) and grade 3/4 thrombocytopenia (OR = 0.50) (32). Recombinant human thrombopoietin (rhTPO), the first-generation agent, is effective but limited by immunogenicity and cost (24–26).

Among second-generation TPO-RAs, romiplostim — a subcutaneous peptibody that binds the native TPO-binding site and additionally exerts immunomodulatory effects — is the best supported for CIT (**Table 2**) (9–12, 27–30). In the phase 3 RECITE trial of 165 patients with persistent CIT, 84% of romiplostim-treated patients needed no CIT-related chemotherapy dose modification versus 36% with placebo (OR 10.16; 95% CI 4.44–23.72; P<0.001) (31); a real-world study likewise showed faster platelet recovery with romiplostim than rhTPO (9 vs. 12 days; P = 0.012) (33).

**Table 2:** Comparison of the thrombopoietin receptor agonists relevant to this case (romiplostim and eltrombopag) and recombinant human thrombopoietin (rhTPO).

Feature	Romiplostim	Eltrombopag	rhTPO
Type	Peptibody (Fc-fusion protein)(s.c.)	Small molecule (Oral)	Recombinant protein (s.c./i.v.)
Administration	Subcutaneous (Weekly)	Oral (Once daily)	Subcutaneous / Intravenous
Mechanism	Binds TPO-R (c-Mpl)	Binds transmembrane domain	Identical to endogenous TPO
Key Features	Long half-life, Immunomodulatory (Treg↑)	Hepatotoxicity risk	First-generation, similar to TPO
Main Indications (Beyond CIT Research)	ITP, CIT, Aplastic Anemia	ITP, Aplastic Anemia, CLD thrombocytopenia	Severe thrombocytopenia (e.g., post-chemo, Hep C)
Dosing Considerations	Weight-based (μg/kg/week) target-based adjustments	Strict: Empty stomach, avoid cations	Variable dose/frequency
Distinct Safety Notes	General TPO-RA risks	Hepatotoxicity, Cataracts (long-term), Strong DDIs	Potential immunogenicity

The oral agents differ mainly in practical terms: eltrombopag requires an empty stomach and carries a higher hepatotoxicity risk (10), avatrombopag has minimal dietary restrictions (9), and hetrombopag is used mainly for adult ITP in China (11). Selection depends on the indication, patient factors, drug interactions and cost, and all require platelet monitoring.

A central question in this case concerns the precise nature of the underlying haematological disorder, and the therapeutic sequence followed the evolution of this diagnostic reasoning. Oral TPO-RAs were used first, consistent with their position as the preferred pharmacological class for CIT; when these failed, a corticosteroid-based immunomodulatory regimen was introduced on the suspicion of secondary, immune-mediated marrow failure; thalidomide and all-trans retinoic acid were selected for their immunomodulatory and megakaryopoiesis-promoting properties, a recognised second-line option for corticosteroid-refractory immune-mediated thrombocytopenia in China (36); rhTPO and, ultimately, romiplostim were added as thrombopoietic support was intensified. Although the bone marrow aspiration (performed at approximately 20 months after diagnosis) showed severe hypocellularity with trilineage suppression and markedly reduced megakaryocytes — morphologically resembling acquired aplastic anaemia (AA) — we regard this case as most consistent with persistent, treatment-related CIT rather than AA, for two reasons. First, a definitive diagnosis of AA could neither be confirmed nor therapeutically tested: the marrow assessment rested on aspiration morphology alone (the comprehensive marrow-failure work-up detailed below was not performed), and standard AA-directed immunosuppression, such as antithymocyte globulin plus ciclosporin, was never administered. Second, and most importantly, the cytopenias did not behave as in AA — the neutropenia and anaemia recovered spontaneously, without lineage-specific treatment, and have remained normal for years, leaving thrombocytopenia as the sole persistent abnormality. This selective, durable single-lineage deficit is more consistent with prolonged, treatment-related bone-marrow and megakaryocytic hypoplasia following high-dose platinum-based chemotherapy combined with 60 Gy thoracic irradiation than with a global stem-cell failure syndrome such as AA. Positioning this case within the broader spectrum of treatment-related bone-marrow failure, it is notable that romiplostim retains activity even when marrow hypoplasia predominates: in refractory AA unresponsive to eltrombopag or to multiple oral TPO-RAs, high-dose romiplostim (initial 20 µg/kg/week) has produced rapid multilineage responses, with some patients maintaining counts after drug discontinuation (34, 35).

Why this patient failed rhTPO yet responded to romiplostim cannot be established from a single case, but a closer look at how the two agents engage c-Mpl suggests several explanations. Activation of c-Mpl requires ligand-induced receptor homodimerisation, and both the efficiency and the duration of that dimerisation influence JAK2 transphosphorylation and the strength of downstream STAT5, PI3K–Akt and MAPK signalling. rhTPO is a recombinant protein carrying the native thrombopoietin sequence and engages the receptor as a single, native ligand molecule. Romiplostim, by contrast, is a peptibody in which four c-Mpl–binding peptides — sharing no sequence homology with endogenous TPO — are displayed on a single immunoglobulin Fc scaffold; this multivalent geometry cross-links receptors more efficiently and may generate a stronger, more complete signal than the native ligand.

Three consequences of this design are relevant here. First, signal duration: expansion and endomitotic maturation of megakaryocyte progenitors depend on sustained rather than pulsatile c-Mpl signalling over several days. FcRn-mediated recycling of the Fc domain gives romiplostim a long half-life, so that weekly injection maintains steady receptor occupancy, whereas rhTPO, even when given at the full standard dose (15000 units/day for 14 days), is rapidly cleared and delivers only intermittent stimulation — plausibly insufficient in a hypoplastic marrow containing few residual progenitors. Second, receptor-mediated clearance: endogenous TPO is removed largely by c-Mpl on platelets and megakaryocytes, so in severe thrombocytopenia with megakaryocytic hypoplasia this clearance pathway is lost and circulating TPO is already high; supplying additional native-sequence ligand may therefore add little, whereas a more potent agonist, or one with more sustained exposure, can still increase net receptor signalling. Third, immunogenicity: because rhTPO is homologous to endogenous TPO, it can elicit neutralising anti-TPO antibodies that abolish the response to further rhTPO — an effect documented chiefly for the early truncated recombinant TPO products, and one that romiplostim, built from non-homologous peptides, was expressly engineered to avoid. Neither anti-TPO antibodies nor TPO levels were measured in this patient, so these mechanisms remain hypotheses. The earlier failure of eltrombopag is separately explained by its distinct binding site (the transmembrane domain rather than the ligand-binding region) and its frequent dose-capping by hepatotoxicity; because it engages a different part of the receptor, its failure does not predict failure of romiplostim.

Several diagnostic limitations should be acknowledged. A comprehensive bone-marrow-failure work-up was not completed: bone-marrow biopsy, flow cytometry, cytogenetic analysis, PNH-clone testing, platelet-antibody testing and molecular studies were not performed. This working classification — most consistent with treatment-related, persistent CIT — therefore rests on the bone-marrow aspiration morphology, the exclusion of malignant infiltration and dysplasia, and the distinctive clinical course described above, rather than on a complete exclusionary panel. As a single-patient observation, this report also precludes mechanistic confirmation; immunophenotyping, cytokine profiling and functional immune assays were not undertaken, and the pharmacological explanations offered above therefore remain hypotheses.

Two issues will require proactive planning in this patient. First, should disease progression necessitate renewed chemotherapy, platelet support must be coordinated in advance, with close monitoring and dose titration before and throughout treatment to keep counts safe and minimise CIT-related delays. Second, should romiplostim lose effect over time, a multi-target approach — for example adding an oral TPO-RA such as avatrombopag, which engages a distinct receptor region — might be explored; this remains, however, a speculative hypothesis, as no clinical cohort or case series has shown that adding an oral TPO-RA after romiplostim failure restores response, and it should be considered only within a research setting (5, 9, 24).

Several practical lessons emerge from this case. First, when chemotherapy-induced thrombocytopenia persists — particularly beyond a few weeks of standard support — early bone-marrow evaluation is advisable to guide therapy, rather than empirically stacking successive agents; in this patient a prolonged refractory interval preceded the marrow assessment that ultimately informed effective treatment. Second, TPO-RA selection may benefit from marrow-guided stratification: patients with marked megakaryocytic hypoplasia and a long transfusion history, as here, may be reasonable candidates for earlier consideration of romiplostim. Third, long-term cancer survivors who develop chronic treatment-related cytopenias require dual, parallel management — ongoing oncological surveillance together with sustained management of treatment toxicity. Finally, dosing should be individualised to identify the minimum effective dose and interval that maintains a safe platelet count, balancing efficacy against patient burden and cost rather than adhering rigidly to a fixed schedule.

In summary, managing CIT within aggressive multimodal SCLC therapy requires strategies that restore platelets without adding toxicity or promoting tumour growth. Romiplostim exemplifies this balance, combining potent thrombopoietic activity with a favourable safety profile, and it enabled durable disease control in this patient. While oral TPO-RAs offer convenience, agent choice should be individualised, and biomarkers to predict CIT risk and guide TPO-RA selection remain an important goal.

## References

[1] Megyesfalvi Z Gay CM Popper H Pirker R Ostoros G Heeke S . Clinical insights into small cell lung cancer: Tumor heterogeneity, diagnosis, therapy, and future directions. CA Cancer J Clin. (2023) 73:620–52. doi: 10.3322/caac.21785 37329269

[2] Cheng Y Spigel DR Cho BC Laktionov KK Fang J Chen Y . Durvalumab after chemoradiotherapy in limited-stage small-cell lung cancer. N Engl J Med. (2024) 391:1313–27. doi: 10.1056/NEJMoa2404873 39268857

[3] Sen T Takahashi N Chakraborty S Takebe N Nassar AH Karim NA . Emerging advances in defining the molecular and therapeutic landscape of small-cell lung cancer. Nat Rev Clin Oncol. (2024) 21:610–27. doi: 10.1038/s41571-024-00914-x 38965396 PMC11875021

[4] Nian Q Liu R Zeng J . Unraveling the pathogenesis of myelosuppression and therapeutic potential of natural products. Phytomedicine. (2024) 132:155810. doi: 10.1016/j.phymed.2024.155810 38905848

[5] Al-Samkari H . Optimal management of chemotherapy-induced thrombocytopenia with thrombopoietin receptor agonists. Blood Rev. (2024) 63:101139. doi: 10.1016/j.blre.2023.101139 37914568 PMC10872905

[6] Wu S Zhang Y Xu L Dai Y Teng Y Ma S . Multicenter, randomized study of genetically modified recombinant human interleukin-11 to prevent chemotherapy-induced thrombocytopenia in cancer patients receiving chemotherapy. Support Care Cancer. (2012) 20:1875–84. doi: 10.1007/s00520-011-1290-x 22041866

[7] Ghanima W Cooper N Rodeghiero F Godeau B Bussel JB . Thrombopoietin receptor agonists: ten years later. Haematologica. (2019) 104:1112–23. doi: 10.3324/haematol.2018.212845 31073079 PMC6545830

[8] Zhang J-J Zhao R Xia F Li Y Wang R-W Guan X . Cost-effectiveness analysis of rhTPO and rhIL-11 in the treatment of chemotherapy-induced thrombocytopenia in hematological tumors based on real-world data. Ann Palliat Med. (2022) 11:2709–19. doi: 10.21037/apm-22-880 36064361

[9] Markham A . Avatrombopag: A review in thrombocytopenia. Drugs. (2021) 81:1905–13. doi: 10.1007/s40265-021-01613-y 34709601 PMC8610948

[10] Corman SL Mohammad RA . Eltrombopag: A novel oral thrombopoietin receptor agonist. Ann Pharmacother. (2010) 44:1072–9. doi: 10.1345/aph.1P042 20460556

[11] Syed YY . Hetrombopag: first approval. Drugs. (2021) 81:1581–5. doi: 10.1007/s40265-021-01575-1 34357499

[12] Soff GA Miao Y Bendheim G Batista J Mones JV Parameswaran R . Romiplostim treatment of chemotherapy-induced thrombocytopenia. J Clin Oncol. (2019) 37:2892–8. doi: 10.1200/JCO.18.01931 31545663 PMC6823892

[13] Lee J Saxena A Giaccone G . Advancements in small cell lung cancer. Semin Cancer Biol. (2023) 93:123–8. doi: 10.1016/j.semcancer.2023.05.008 37236329

[14] Petty WJ Paz-Ares L . Emerging strategies for the treatment of small cell lung cancer: A review. JAMA Oncol. (2023) 9:419. doi: 10.1001/jamaoncol.2022.5631 36520421

[15] Bogart JA Waqar SN Mix MD . Radiation and systemic therapy for limited-stage small-cell lung cancer. J Clin Oncol. (2022) 40:661–70. doi: 10.1200/JCO.21.01639 34985935 PMC10476774

[16] Mei J Jiao F Li Y Cui J Yang H Wang L . Application of thrombopoietic agents in cancer therapy-induced thrombocytopenia: A comprehensive review. Blood Rev. (2025) 70:101257. doi: 10.1016/j.blre.2025.101257 39809679

[17] Giffin MJ Cooke K Lobenhofer EK Estrada J Zhan J Deegen P . AMG 757, a half-life extended, DLL3-targeted bispecific T-cell engager, shows high potency and sensitivity in preclinical models of small-cell lung cancer. Clin Cancer Res. (2021) 27:1526–37. doi: 10.1158/1078-0432.CCR-20-2845 33203642

[18] Faivre-Finn C Snee M Ashcroft L Appel W Barlesi F Bhatnagar A . Concurrent once-daily versus twice-daily chemoradiotherapy in patients with limited-stage small-cell lung cancer (CONVERT): An open-label, phase 3, randomised, superiority trial. Lancet Oncol. (2017) 18:1116–25. doi: 10.1016/S1470-2045(17)30318-2 28642008 PMC5555437

[19] Epstein RS Weerasinghe RK Parrish AS Krenitsky J Sanborn RE Salimi T . Real-world burden of chemotherapy-induced myelosuppression in patients with small cell lung cancer: A retrospective analysis of electronic medical data from community cancer care providers. J Med Econ. (2022) 25:108–18. doi: 10.1080/13696998.2021.2020570 34927520

[20] Bhatia M Davenport V Cairo MS . The role of interleukin-11 to prevent chemotherapy-induced thrombocytopenia in patients with solid tumors, lymphoma, acute myeloid leukemia and bone marrow failure syndromes. Leuk Lymphoma. (2007) 48:9–15. doi: 10.1080/10428190600909115 17325843

[21] Huynh J Baloyan D Chisanga D Shi W O’Brien M Afshar-Sterle S . Host IL11 signaling suppresses CD4+ T cell–mediated antitumor responses to colon cancer in mice. Cancer Immunol Res. (2021) 9:735–47. doi: 10.1158/2326-6066.CIR-19-1023 33906864

[22] Xiong W Chen Y Zhang C Li J Huang H Zhu Y . Pharmacologic inhibition of IL11/STAT3 signaling increases MHC-I expression and T cell infiltration. J Transl Med. (2023) 21:416. doi: 10.1186/s12967-023-04079-6 37365574 PMC10294373

[23] Heichler C Scheibe K Schmied A Geppert CI Schmid B Wirtz S . STAT3 activation through IL-6/IL-11 in cancer-associated fibroblasts promotes colorectal tumour development and correlates with poor prognosis. Gut. (2020) 69:1269–82. doi: 10.1136/gutjnl-2019-319200 31685519

[24] Song AB Al-Samkari H . Emerging data on thrombopoietin receptor agonists for management of chemotherapy-induced thrombocytopenia. Expert Rev Hematol. (2023) 16:365–75. doi: 10.1080/17474086.2023.2201428 37039010 PMC10190112

[25] Liu X Bai Y Wang T Song Y Sun F Xia R . Recombinant human thrombopoietin (rhTPO) of different dosing regimens for refractory/relapsed primary immune thrombocytopenia: A multicenter, randomized controlled trial and pharmacokinetics study. Platelets. (2023) 34:2157806. doi: 10.1080/09537104.2022.2157806 36597010

[26] Luan H Yang J Wang Y Shen X Zhang X Qiao Z . rhTPO ameliorates radiation-induced long-term hematopoietic stem cell injury in mice. Molecules. (2023) 28:1953. doi: 10.3390/molecules28041953 36838940 PMC9961369

[27] Song AB Al‐Samkari H . Chemotherapy‐induced thrombocytopenia: Modern diagnosis and treatment. Br J Haematol. (2025) 206:1062–6. doi: 10.1111/bjh.20037 40040262 PMC12140172

[28] Bussel JB Soff G Balduzzi A Cooper N Lawrence T Semple JW . A review of romiplostim mechanism of action and clinical applicability. Drug Des Devel Ther. (2021) 15:2243–68. doi: 10.2147/DDDT.S299591 34079225 PMC8165097

[29] Soff GA Al‐Samkari H Leader A Eisen M Saad H . Romiplostim in chemotherapy‐induced thrombocytopenia: A review of the literature. Cancer Med. (2024) 13:e7429. doi: 10.1002/cam4.7429 39135303 PMC11319220

[30] Schifferli A Rüfer A Rovo A Nimmerjahn F Cantoni N Holbro A . Immunomodulation with romiplostim as a second‐line strategy in primary immune thrombocytopenia: The iROM study. Br J Haematol. (2023) 203:119–30. doi: 10.1111/bjh.19074 37735543

[31] Al-Samkari H Munoz C Geredeli C Korantzis I Gonzalez Astorga B Arslan C . Romiplostim versus placebo for chemotherapy-induced thrombocytopenia. N Engl J Med. (2026) 394:1061–73. doi: 10.1056/NEJMoa2511882 41812193

[32] Yang H Xu X Tan M Gao J Fang R Liu X . Efficacy and safety of different treatments in chemotherapy-induced thrombocytopenia: A systematic review and network meta-analysis. Front Pharmacol. (2025) 16:1549214. doi: 10.3389/fphar.2025.1549214 40766766 PMC12321752

[33] Cheng Y Wei J Zhu F Xuan L Li Y Meng L . Comparison of the real-world efficacy and safety of romiplostim and recombinant human thrombopoietin (rhTPO) in chemotherapy-induced thrombocytopenia. BMC Cancer. (2026) 26:320. doi: 10.1186/s12885-026-15678-z 41629833 PMC12958640

[34] Hosokawa K Yamazaki H Tanabe M Sugimori N Takamatsu H Nakao S . High-dose romiplostim accelerates hematologic recovery in patients with aplastic anemia refractory to eltrombopag. Leukemia. (2021) 35:906–9. doi: 10.1038/s41375-020-0950-6 32616921

[35] Lin X Yang C Liu Z Zhuang J Chen M Han B . Effective treatment of refractory aplastic anemia with romiplostim after failure of multiple thrombopoietin receptor agonists: A single-center retrospective study. Ann Med. (2025) 57:2514791. doi: 10.1080/07853890.2025.2514791 40534403 PMC12180318

[36] Huang Q-S Liu Y Wang J-B Peng J Hou M Liu H . All-trans retinoic acid plus high-dose dexamethasone as first-line treatment for patients with newly diagnosed immune thrombocytopenia: A multicentre, open-label, randomised, controlled, phase 2 trial. Lancet Haematol. (2021) 8:e688–99. doi: 10.1016/S2352-3026(21)00240-4 34560012

